# From Hernia to Urachal Remnant: A Report of an Unusual Case of a Young Adult

**DOI:** 10.7759/cureus.90063

**Published:** 2025-08-14

**Authors:** Camila Santos, Jhon Coronel, Luis Dorado

**Affiliations:** 1 Surgery, Hospital General Monte Sinaí, Guayaquil, ECU

**Keywords:** adult urachal sinus, general surgery, infected urachal sinus, infection, urachal remnant

## Abstract

Urachal sinus is an uncommon condition in the adult population. It is most often diagnosed incidentally in children and typically presents with symptoms in adults only when associated with infection. The clinical presentation is nonspecific, frequently leading to misdiagnosis as other abdominal pathologies. Current literature supports the use of antibiotic therapy followed by complete surgical resection of the urachal sinus due to its potential risk of progression to bladder adenocarcinoma. This report presents a case of an adult patient with abdominal pain and umbilical discharge who was ultimately diagnosed with a urachal remnant based on histopathological findings. Following appropriate antibiotic therapy and complete surgical excision, the patient showed marked clinical improvement.

## Introduction

The urachus is a remnant of the allantois that develops into a tubular structure during the third week of gestation, connecting the ventral cloaca to the yolk sac. During embryological life, the urachus lies between the transversalis fascia and the parietal peritoneum, extending from the apex of the bladder to the umbilicus. Under normal conditions, the urachus undergoes complete regression between the 26th and 28th weeks of gestation, eventually forming the median umbilical ligament [1]. However, in some cases, incomplete obliteration can result in persistent urachal anomalies such as a sinus, diverticulum, cyst, or a completely patent urachus [2].

Urachal remnants are found in approximately 1.03% of children, with a male-to-female ratio of 2:1. They are frequently discovered incidentally during radiological examinations or exploratory laparotomies [3]. Symptomatic presentation usually occurs between 20 and 40 years of age, primarily due to secondary infection of the tubular remnants [4]. Although diagnosis is often made through imaging studies, contrast-enhanced computed tomography is considered the gold standard for definitive evaluation [5].

The treatment of infected urachal remnants typically involves antibiotic therapy to manage the acute infection, followed by surgical excision. A laparoscopic approach is recommended as it is a safe and effective procedure [6]. Surgical intervention is necessary due to the risk of recurrent infections, which occur in approximately 30% of cases, as well as the potential risk of adenocarcinoma development [7].

## Case presentation

We report the case of a 25-year-old male patient with no significant past medical history who presented to the hospital with a 15-day history of abdominal symptoms. The clinical picture was characterized by umbilical pain radiating to both the right and left hypochondrium, associated with undocumented fever and purulent umbilical discharge.

**Figure 1:**
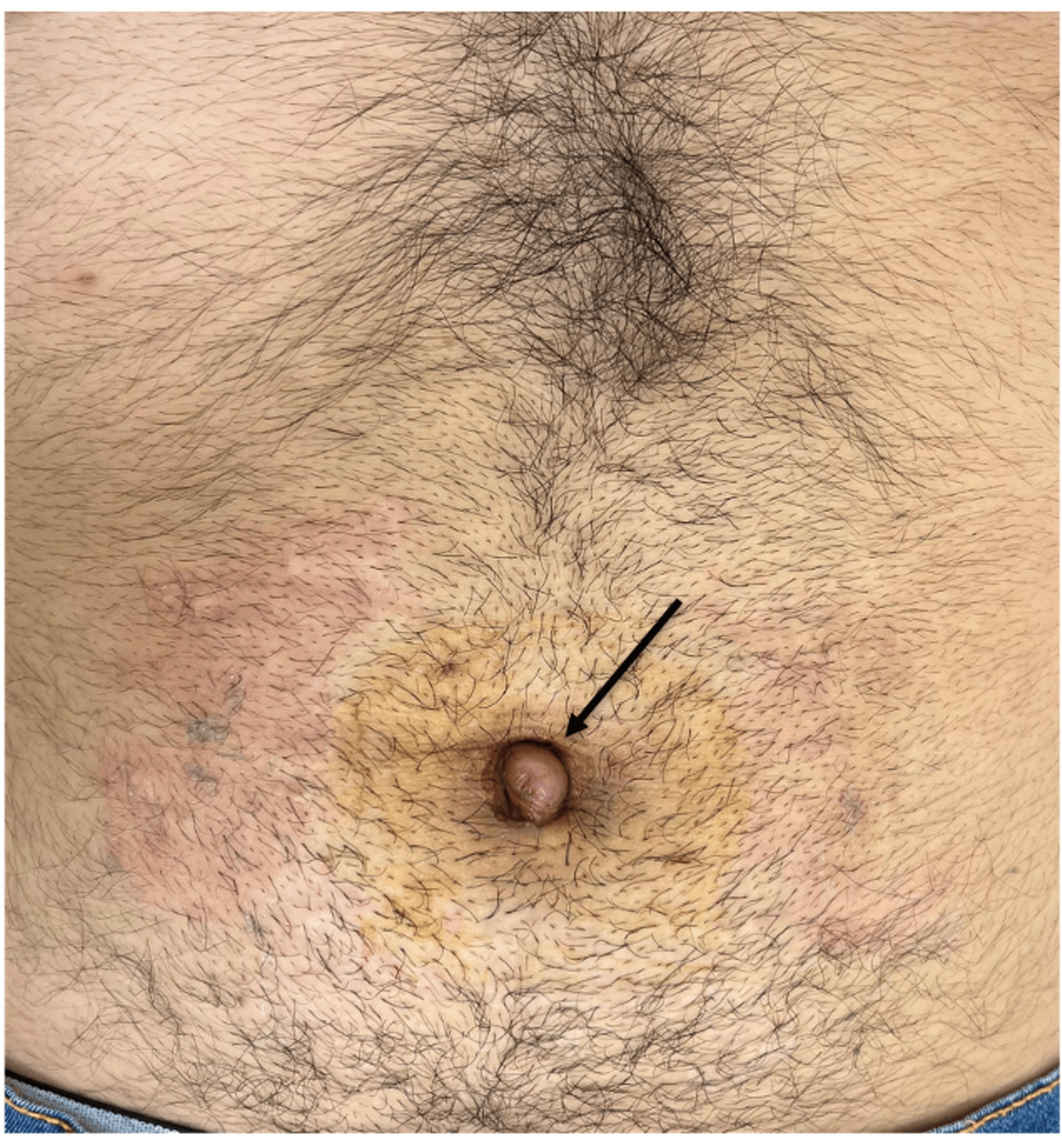
Physical examination: umbilical region showing protrusion of the navel

On physical examination, the umbilical region showed noticeable protrusion and erythema. Palpation revealed no evidence of a hernial ring (Figure 1).

**Figure 2:**
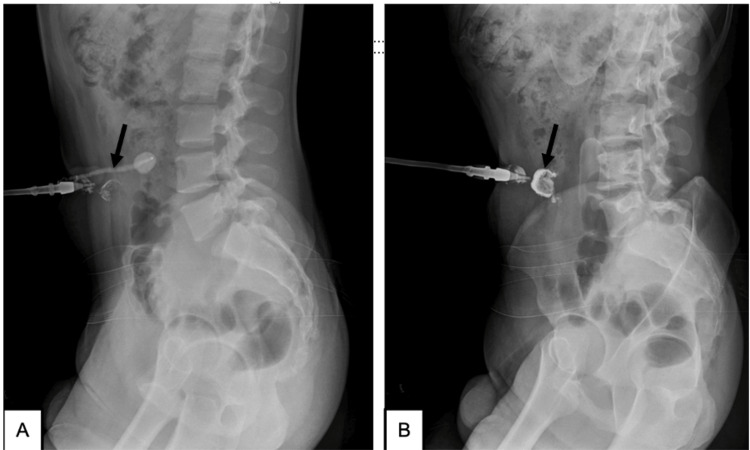
Fistulogram: A and B show an infraumbilical fistulous tract

A fistulogram was performed, revealing an infraumbilical fistulous tract with no apparent communication with the bladder (Figure 2).

**Figure 3:**
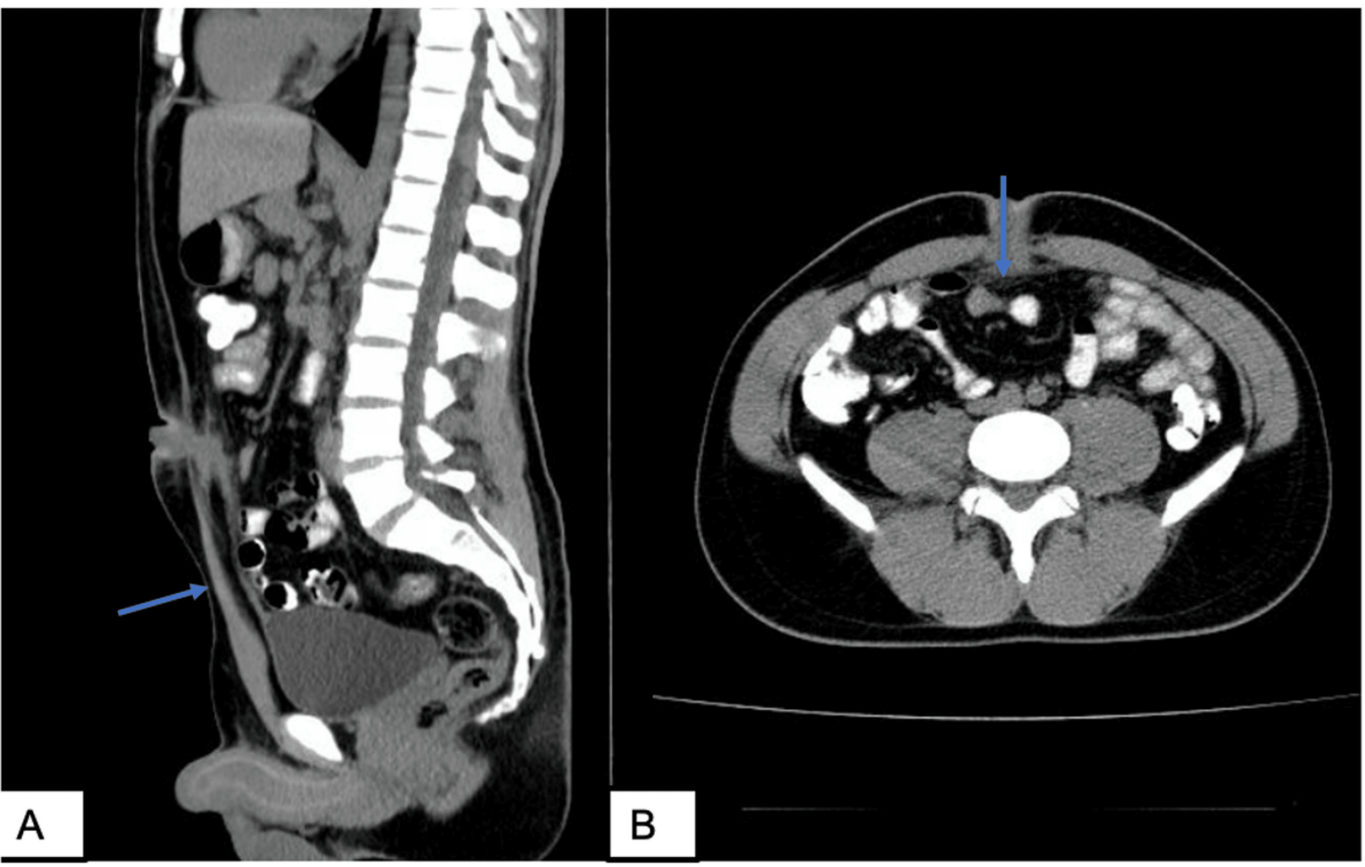
Contrast-enhanced abdominal and pelvic CT scan: A. Sagittal view: Hypodense tubular structure extending from the bladder dome to the anterior abdominal wall. B. Axial view: Hypodense structure with surrounding mesenteric fat stranding.

Based on the findings consistent with a urachal fistula, a contrast-enhanced computed tomography (CT) scan of the abdomen and pelvis was performed. The CT revealed a hypodense tubular structure extending from the dome of the bladder to the anterior abdominal wall along the midline. The structure contained both fluid and gas. Additionally, the axial view showed surrounding mesenteric fat stranding (Figure 3).

Given the diagnosis of a urachal malformation with a suspected infectious focus, intravenous antibiotic therapy with cefalexin 1 gram daily was initiated. Surgical intervention was then decided, consisting of urachal excision, omphalectomy, and omphaloplasty.

An open surgical approach was selected to ensure complete resection of the urachal remnant under direct visualization. In addition, the case was performed in conjunction with the plastic surgery team, who carried out simultaneous umbilical reconstruction (neoumbilicoplasty), which was more easily facilitated through an open infraumbilical incision.

**Figure 4:**
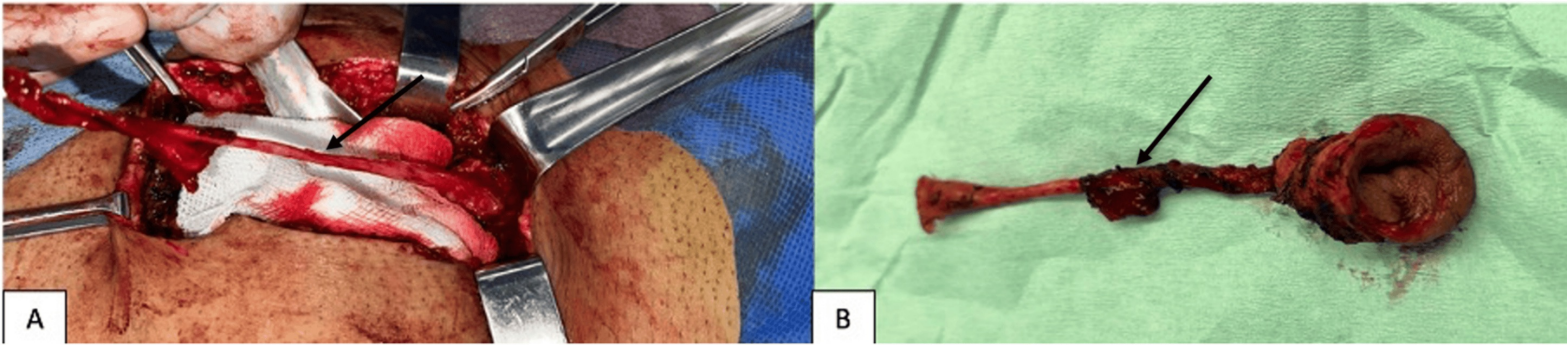
Intraoperative findings: A) Fibrous cord extending from the umbilical region to the bladder dome. B) Umbilical fistula with necrotic tissue.

An exploratory laparotomy was performed through a midline infraumbilical incision of approximately 20 cm, proceeding layer by layer until reaching the supraperitoneal space. In the deep planes, a tubular structure running in the midline and connecting the umbilicus to the bladder dome was identified, consistent with a persistent urachus and with a fibrous appearance. Careful dissection of the surrounding tissues was carried out, achieving complete isolation of the structure, followed by total excision. An en bloc omphalectomy was performed. At the bladder level, the dome was closed in two layers. Methylene blue was introduced through the urinary catheter to test for watertightness, confirming the absence of leakage. Intraoperatively, a partially obliterated fibrous cord was observed, extending from the umbilical region to the bladder dome. Additionally, an umbilical fistula with necrotic tissue was identified (Figure 4).

Subsequently, the Plastic Surgery Service performed a neoumbilicoplasty for umbilical reconstruction. The abdominal midline was closed anatomically in layers up to the skin.

**Figure 5:**
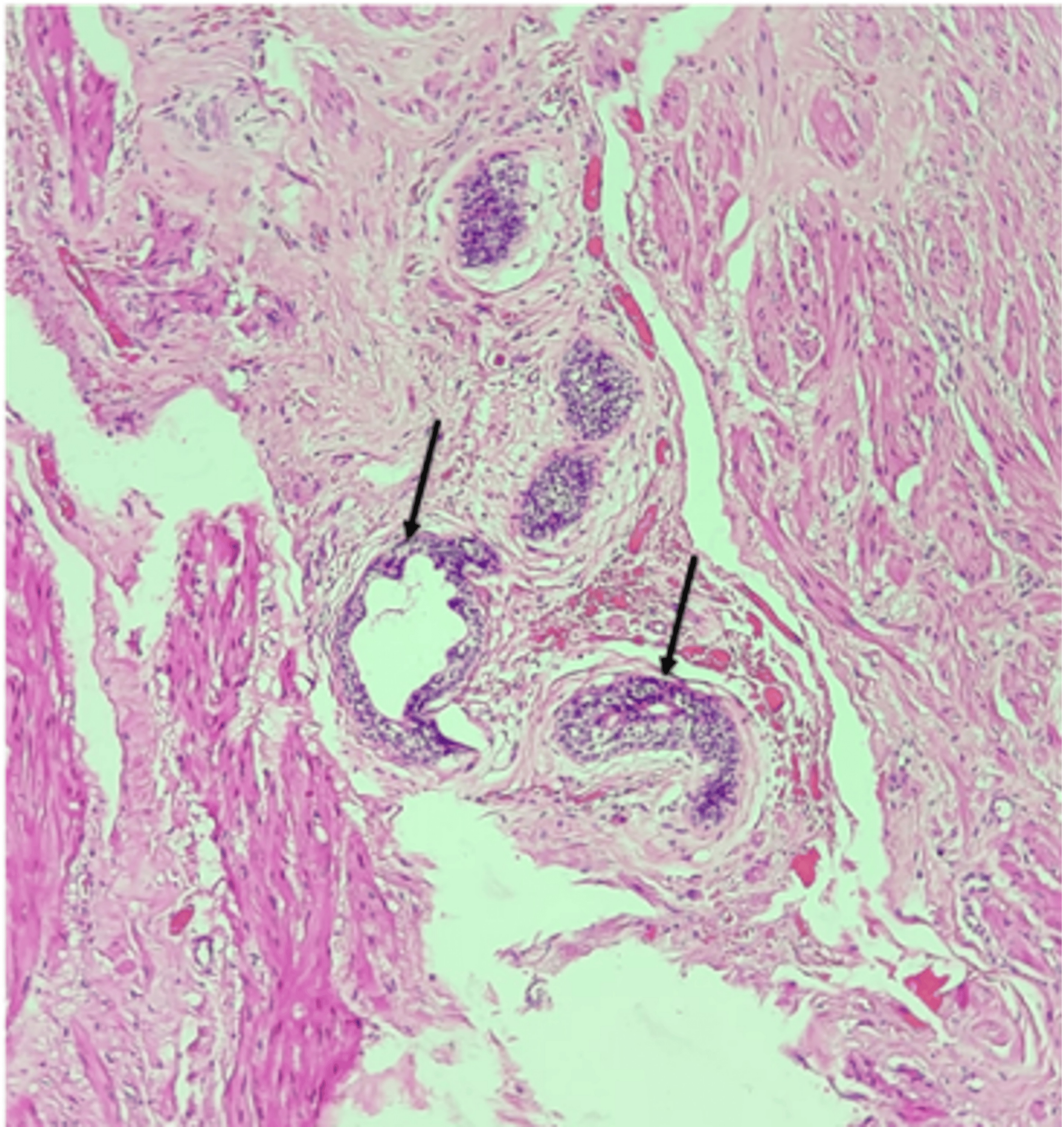
Histopathological section stained with H&E: tubular structure lined by columnar or cuboidal epithelium, featuring a clear lumen, orderly arranged cells, and areas of metaplastic epithelium

The surgical specimen was sent for histopathological examination. Hematoxylin and eosin staining revealed connective tissue with dilated and congested blood vessels. Additionally, a tubular structure lined by columnar or cuboidal epithelium with a clear lumen and orderly arranged cells was observed. This structure was surrounded by fibrous connective tissue and areas of metaplastic epithelium. These findings support the diagnosis of a urachal malformation (Figure 5).

**Figure 6:**
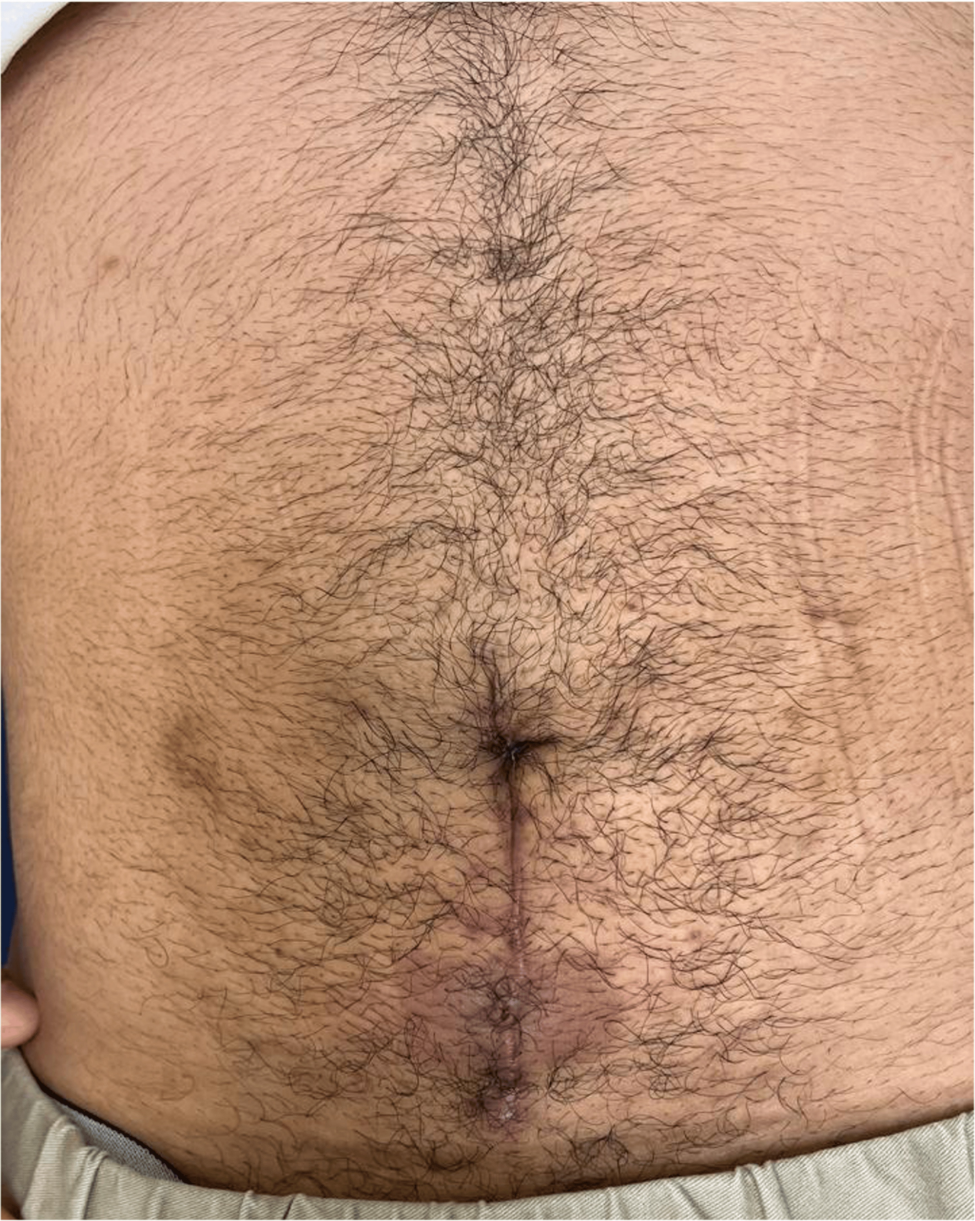
Six-month follow-up

Following surgery, the patient demonstrated significant clinical improvement with resolution of purulent umbilical discharge. The postoperative course was uneventful, and he was discharged in stable condition three days after the procedure. A six-month follow-up was performed, during which the patient showed no complications and complete resolution of symptoms (Figure 6).

## Discussion

Urachal remnants are relatively uncommon in adults worldwide, with an estimated incidence of approximately 1 in 1500 individuals. In a retrospective study by Ashley et al., one of the largest studies focusing on urachal anomalies in adults, 176 patients with urachal remnants were identified [8]. Among these, 130 were adults (86 males and 44 females), with a median age of 54.5 years. In our case, the patient was a 25-year-old male, which is notable given that urachal remnants are more frequently reported in males, further supporting the sex distribution observed in the literature.

In a retrospective study by Risher et al., 41 cases of urachal anomalies in adults were identified [9]. Among these, urachal cysts were the most common (12.19%), followed by urachal sinuses (9.75%), vesicourachal diverticula (2.43%), and alternating sinuses (2.43%). In our case, the diagnosis was a urachal sinus, highlighting the importance of considering this rare anomaly in the differential diagnosis, despite its low incidence in the adult population.

The clinical presentation of urachal anomalies in adults often differs from that in children. In pediatric populations, these anomalies are typically asymptomatic and frequently identified as incidental findings. However, when symptoms are present, they tend to manifest later in life. In adults, urachal anomalies may present with umbilical pain or more nonspecific symptoms such as abdominal discomfort, hematuria, dysuria, urinary frequency, or urgency. Among these anomalies, infection is the most common complication [10]. In a retrospective study by Gopalan et al., nine patients with infected urachal remnants were identified [11]. The most common presenting symptom was abdominal pain (88.8%), followed by fever (66.6%), while umbilical discharge was reported less frequently.

In the present case, the initial clinical suspicion was an umbilical hernia. However, following a more comprehensive medical history, this diagnosis was ruled out. The patient presented with umbilical pain and purulent drainage, raising suspicion for an underlying infection. This case underscores the importance of a detailed clinical evaluation in distinguishing urachal anomalies from other conditions with similar presentations.

The diagnosis of urachal anomalies can be achieved using various imaging modalities, including ultrasound (US), computed tomography (CT), and/or magnetic resonance imaging (MRI) [12]. In the present case, a fistulogram, combined with contrast-enhanced abdominal and pelvic CT, was performed. This approach provided sufficient detail to accurately identify the presence of a urachal remnant, underscoring the value of CT imaging in the diagnostic workup of such anomalies.

The treatment of choice following suspicion of an infection associated with a urachal remnant is the administration of appropriate antibiotic therapy followed by complete surgical excision [13]. Incomplete resection has been associated with a recurrence rate of approximately 30%. Moreover, radical excision is recommended given that urachal anomalies are believed to carry an increased risk of bladder adenocarcinoma in adults [14]. Urachal adenocarcinoma, although rare, has an estimated incidence of 0.18 per 100,000 individuals per year [15]. In the present case, histopathological examination revealed no evidence of malignancy.

This case reinforces the value of a multidisciplinary approach and emphasizes the need to consider even uncommon pathologies in the adult population when clinical findings are atypical. Although rare, urachal remnants can present with signs that mimic more common conditions such as umbilical hernias or soft tissue infections. This case highlights the importance of maintaining a high index of suspicion for urachal anomalies in adult patients presenting with umbilical symptoms. 

## Conclusions

The urachal remnant in adults is a condition with a low global incidence. However, it should be considered in patients presenting with fever, abdominal pain, umbilical protrusion, and umbilical discharge. Early diagnosis and treatment help prevent major complications. This case highlights the importance of considering other differential diagnoses with similar symptoms, so that with appropriate therapy (complete surgical resection), a favorable clinical outcome can be achieved. Nonetheless, diagnosis remains challenging in the early stages of the condition.
